# Editorial: Host-Pathogen Interactions During Arboviral Infections

**DOI:** 10.3389/fcimb.2019.00077

**Published:** 2019-03-26

**Authors:** Alan G. Goodman, Angela L. Rasmussen

**Affiliations:** ^1^School of Molecular Biosciences, College of Veterinary Medicine, Washington State University, Pullman, WA, United States; ^2^Paul G. Allen School for Global Animal Health, College of Veterinary Medicine, Washington State University, Pullman, WA, United States; ^3^Center for Infection and Immunity, Columbia University Mailman School of Public Health, New York, NY, United States

**Keywords:** West Nile virus, dengue virus, chikungunya virus, tick-borne encephalitis virus, Japanese encephalitis virus, Zika virus, systems biology, immunity

Arboviruses (Arbo = ARthropod-BOrne) are a diverse group of viruses that are transmitted by arthropod vectors, most commonly by insects such as mosquitoes and blood-feeding flies, or arachnids such as ticks. With the exception of African swine fever virus, a DNA virus, all arboviruses are RNA viruses and belong to one of six viral families: *Bunyaviridae* (e.g., Rift Valley fever virus), *Reoviridae* (e.g., Bluetongue virus), *Orthomyxoviridae* (e.g., Bourbon virus), *Rhabdoviridae* (e.g., vesicular stomatitis virus), *Togaviridae* (e.g., equine encephalitis and chikungunya viruses), or *Flaviviridae* (e.g., tick-borne encephalitis, dengue, Japanese encephalitis, West Nile, Zika, and yellow fever viruses) (Hanley and Weaver, 2008). Flaviviruses pose a major human threat, and the world's population is at risk of infection with several flaviviruses: In the United States, West Nile virus (WNV) is the leading cause of arbovirus infections (Lindsey et al., 2014) and caused over 2,000 confirmed cases in 2017 (ArboNET, 2018). Zika virus (ZIKV), while previously considered a minimal threat to human health, is now present in over 30 countries (Tham et al., 2018). Additionally, over 40% of the world's population is at risk for dengue virus (DENV) infection (Jindal et al., 2014). All of these flaviviruses can cause severe complications in human patients: two-thirds of West Nile virus cases are classified as neuroinvasive, Zika virus is associated with Guillain-Barré syndrome and microcephaly, and dengue virus causes hemorrhagic fever in 500,000 patients annually. There are no post-exposure therapeutics available for any flavivirus and no approved vaccines other than for yellow fever and Japanese encephalitis viruses, indicating an unmet need in medicine. Moreover, diabetic individuals are at a higher risk for contracting West Nile virus disease (Nash et al., 2001). Flavivirus infection is only expected to increase, due to climate change affecting the geographical ranges of the mosquito vectors (Chen et al., 2013; Kraemer et al., 2015). Thus, it is imperative that current research investigates the both the immune response to and pathogenesis of these arboviral infections. The purpose of this research topic is to provide a platform for the dissemination of high-quality primary research articles, comprehensive reviews, and opinions that explore the host responses to arboviral infections. Serendipitously, each of the five review articles published in our research topic nicely introduces one of the five primary research articles, and our topic includes an opinion article that discusses flaviviral vaccine development, which suitably ties together the research topic.

An important topic covered is the growing threat of infection by tick-borne viral pathogens. Mlera and Bloom highlight the need for further research into the role of small-to-medium-sized mammals in tick-borne flavivirus (TBFV) biology. Rodents are a true reservoir of TBFVs, as these animals harbor the virus without showing clinical disease. While the research community agrees that rodents play a major role in TBFV transmission, there remains a need for better understanding of the host response to infection with these viruses. TBFV infections in humans are often neurotropic and can cause acute neuroinflammation or tick-borne encephalitis (TBE) disease. Cases of TBE are highest in the summer-autumn period, and Daniel et al. provide epidemiological analysis arguing that this is likely caused by increased viral replication at higher temperatures. Thus, bites during the summer-autumn period deliver a higher viral load to humans, and they call for these data to be used to forecast TBE risk.

Mosquito-borne viruses and the host responses that determine tropism and pathogenicity are also covered. As reviewed by Ahlers and Goodman, while *Culex* mosquitoes transmit WNV to dead-end mammalian hosts, birds are reservoirs for WNV. Each host species exhibits similar innate immune responses that function through detection of viral RNA and subsequent JAK/STAT pathway activation that leads to adaptive immunity in mammals. In humans, WNV begins replicating in the skin at the site of mosquito bite before traveling to the lymph node, which can lead to viremia and neuronal infections. Garcia et al. show that keratinocytes exhibit a type I and III interferon response and elevated pro-inflammatory cytokines when infected. Interestingly, the addition of mosquito saliva to keratinocytes reduced WNV-mediated inflammatory responses and lead to increased viral replication.

Key host cellular mechanisms of pathogenesis are explored, including the important role of host lipids in viral replication and downstream effects on specific host tissues. Cholesterol plays a fundamental role during flavivirus infection, from viral entry and innate immune responses to viral egress. Due to the dependence of flaviviruses on cholesterol, Osuna-Ramos et al. contend that FDA-approved cholesterol-lowering drugs could be repurposed to combat flaviviral infections. Cholesterol and other lipid molecules also play a major role in epidermal homeostasis and hair follicle regeneration (Driskell et al., 2014). Here, Wei et al. show that DENV infects dermal papilla cells, leading to reduced cell viability due to increased inflammatory cytokines and caspase activation. These results could mechanistically explain why some DENV-infected individuals display hair loss as a clinical manifestation.

Since the year 2000, computational and mathematical modeling of biological systems, also known as systems biology, has been widely used as a holistic approach to discover novel ways in which the virus and host interact. Here, Petit and Shah review how systems biology, particularly next-generation sequencing, mass spectrometry, and other “-omics”-based techniques, have been used in the study of arbovirus-vector interactions. For example, Chan et al. performed a kinase/phosphatase-wide RNAi screen to discover that checkpoint kinase 2 (CHK2), which regulates the cell cycle, is activated during Japanese encephalitis virus (JEV) infection to aid in its replication. As such, inhibition or knockdown of CHK2 reduced JEV infection. This study elegantly demonstrates how systems biology is used to identify new strategies to combat arboviral infections.

Chikungunya virus (CHIKV) is a re-emerging mosquito-borne arbovirus that has caused several epidemics throughout the world in the last decade. Tanabe et al. stress the importance of fully understanding the host cellular response in order to discover new soluble markers of infection and therapeutic strategies, including new vaccines and monoclonal antibodies, to diagnose and combat viral disease. With regard to cellular markers and tropism of flaviviral infections, García-Nicolás et al. used porcine and human monocyte-derived dendritic cells to test flavivirus infectivity. They demonstrated that select viruses interfered with cytokine production and innate immunity in a species-dependent manner. Together, these articles emphasize the significance of cellular responses in realizing the zoonotic and pathogenic potential of arboviral infection.

Finally, Martín-Acebes et al. explore the phenomenon of antibody-dependent enhancement (ADE), which may lead to disease exacerbation during ZIKV infection. Specifically, the development of immunity to other flaviviral infections, particularly DENV, with which ZIKV co-circulates, or yellow fever virus, for which there is a vaccine, could facilitate ADE during ZIKV infection. However, more epidemiological and animal model data must be analyzed before fully understanding the role of ADE during ZIKV infection and pathogenesis. The findings from these studies could also help combat infection with each of the other arboviruses discussed in this research topic.

As ecological change drives the spread of vector species into new geographical regions and incidence increases, arboviral pathogens are a growing threat to public health. Research that characterizes host-pathogen interactions and mechanisms of pathogenesis is critically important to understanding the underlying biology and developing effective medical countermeasures. In conclusion, we want to thank all authors and reviewers for their valuable contributions and insight to this research topic. This topic is timely and we hope that it inspires increased research efforts and insight into arbovirus biology and pathogenicity to ultimately alleviate the burden of arboviral infections.

## Author Contributions

AG wrote the first draft of the manuscript. AR revised the manuscript.

### Conflict of Interest Statement

The authors declare that the research was conducted in the absence of any commercial or financial relationships that could be construed as a potential conflict of interest.
